# Deubiquitinating Enzymes in Alzheimer’s Disease: From Protein Homeostasis to Diverse Pathological Processes

**DOI:** 10.3390/biom16101494

**Published:** 2026-10-14

**Authors:** Xueying Shi, Guilin Chen, Xiufeng Wang, Rong Ma, Gang Li

**Affiliations:** 1 Department of Neurology, Union Hospital, Tongji Medical College, Huazhong University of Science and Technology, Wuhan 430022, China; 2 Department of Pharmacology, School of Basic Medicine, Tongji Medical College, Huazhong University of Science and Technology, Wuhan 430022, China

**Keywords:** Alzheimer’s disease, deubiquitinating enzymes, ubiquitination, proteostasis, autophagy, neuroinflammation

## Abstract

Alzheimer’s disease (AD) is a neurodegenerative disorder involving multiple pathological processes, including amyloid-β (Aβ) accumulation, tau pathology, impaired autophagy, mitochondrial dysfunction, neuroinflammation, and synaptic dysfunction. Deubiquitinating enzymes (DUBs) reverse ubiquitination and regulate protein stability, degradation, and cellular signaling. Although studies of DUBs in AD have largely focused on Aβ metabolism and tau homeostasis, accumulating evidence implicates DUBs in a broader range of AD-related processes, including autophagy, neuroinflammation, mitochondrial function, and synaptic function. Several DUBs have also been implicated in more than one of these processes, although these effects have often been identified in different experimental models. In this review, we summarize current knowledge of DUBs in major AD-related pathological processes and discuss the mechanisms through which individual DUBs may influence different aspects of AD pathology. We also discuss the limitations of current studies and the potential and challenges of targeting DUBs in AD.

## 1. Introduction

Alzheimer’s disease (AD) is the most common neurodegenerative disease and the leading cause of dementia. Its clinical manifestations are characterized primarily by progressive cognitive decline and memory impairment [1]. The defining pathological hallmarks of AD are extracellular amyloid plaques formed by amyloid-β (Aβ) deposition and intracellular neurofibrillary tangles composed of abnormally phosphorylated tau. These hallmark lesions are accompanied by other pathological alterations, including neuroinflammation, mitochondrial dysfunction, impaired autophagy, and synaptic damage [2]. The persistent accumulation of abnormal proteins such as Aβ and tau reflects disrupted proteostasis in the AD brain [3]. In addition to increased production of these proteins, dysfunction of protein degradation and clearance systems contributes to the accumulation of misfolded and aggregated proteins, thereby promoting AD progression.

The ubiquitin-proteasome system is a major component of cellular protein quality control. Ubiquitination is a highly dynamic post-translational modification mediated by the sequential actions of E1 ubiquitin-activating enzymes, E2 ubiquitin-conjugating enzymes, and E3 ubiquitin ligases. This enzymatic cascade attaches ubiquitin to substrate proteins and thereby regulates their stability, localization, activity, and interactions [4]. Ubiquitin chains can be assembled through eight canonical linkage types, namely M1, K6, K11, K27, K29, K33, K48, and K63, which encode diverse cellular signals [5]. Among these, K48-linked polyubiquitin chains generally serve as signals for proteasomal degradation, whereas K63-linked chains predominantly participate in non-proteolytic processes, including protein trafficking and cellular signaling [6]. Ubiquitination therefore functions not only as a signal for protein degradation but also as a versatile regulatory system that determines protein fate and influences diverse cellular processes.

**Figure 1:**
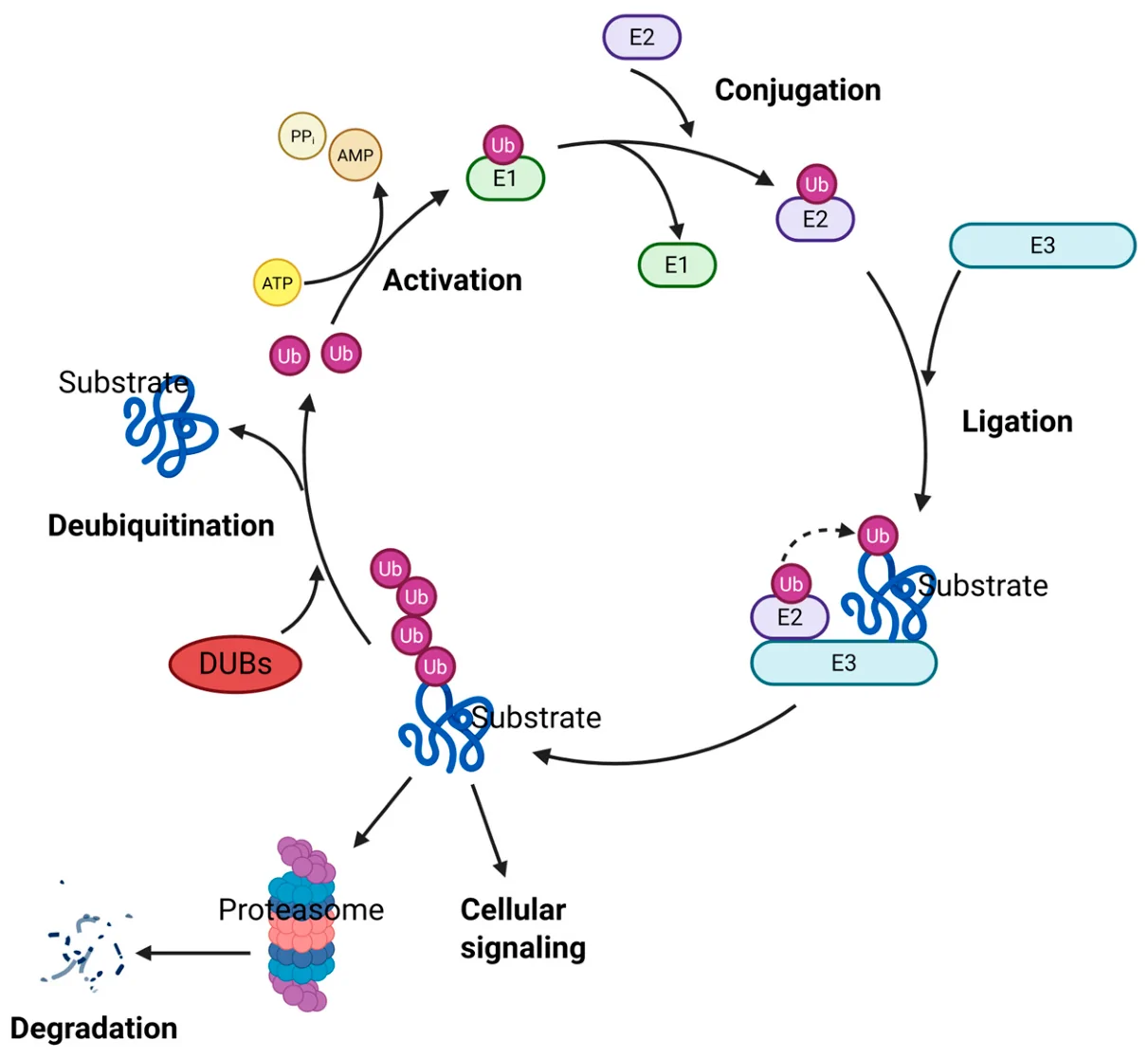
Schematic overview of ubiquitination and deubiquitination. Ubiquitination involves the sequential action of E1, E2, and E3 enzymes, which attach ubiquitin (Ub) to substrate proteins. Depending on the type of ubiquitin modification, ubiquitinated proteins may undergo proteasomal degradation or participate in cellular signaling pathways. DUBs remove ubiquitin from substrates or edit ubiquitin chains, thereby regulating substrate stability and allowing ubiquitin recycling.

Ubiquitination is reversible, and its reversal is mediated by deubiquitinating enzymes (DUBs). By removing ubiquitin from substrate proteins or editing ubiquitin chains, DUBs regulate protein stability and function and contribute to the maintenance of cellular proteostasis [7] (Figure 1). Growing evidence indicates that the roles of DUBs in AD extend beyond the regulation of Aβ metabolism and tau homeostasis to autophagy, mitochondrial function, neuroinflammation, and synaptic function. For example, USP9X may exacerbate tau pathology by suppressing autophagy [8], whereas USP25 promotes neuroinflammation and aggravates AD-related pathology [9]. Previous reviews have focused primarily on the regulation of Aβ and tau by DUBs [10], whereas evidence for the broader involvement of DUBs in other AD-related pathological processes remains scattered and has not been comprehensively evaluated. In this review, we summarize the roles and molecular mechanisms of DUBs in Aβ and tau pathology, autophagic dysfunction, mitochondrial dysfunction, neuroinflammation, synaptic dysfunction, and other AD-related processes. We further discuss DUBs that act across multiple pathological processes, critically evaluate the context and limitations of current evidence, and consider the therapeutic opportunities and challenges associated with modulating DUB activity in AD.

## 2. Classification of DUBs and Their Dysregulation in Alzheimer’s Disease

Deubiquitinating enzymes (DUBs) are proteases that reverse or edit ubiquitination by cleaving ubiquitin chains or removing ubiquitin from substrate proteins. Through these activities, DUBs regulate proteostasis, cellular signaling, cell-cycle progression, and DNA damage responses. The human genome encodes approximately 100 DUBs, which are classified into seven major families according to their catalytic domains. The ubiquitin-specific protease (USP), ubiquitin C-terminal hydrolase (UCH), ovarian tumor protease (OTU), Machad–Josephin domain-containing protease (MJD), MINDY, and ZUFSP/ZUP1 families are cysteine proteases whose catalytic activity depends on an active-site cysteine residue. In contrast, the JAMM/MPN+ family comprises zinc-dependent metalloproteases and is the only DUB family that does not use a cysteine-based catalytic mechanism [7]. Differences in domain architecture, substrate recognition, and ubiquitin-chain selectivity underlie the functional diversity of DUBs.

Accumulating evidence suggests that ubiquitin homeostasis is disrupted in AD [11]. As key regulators of ubiquitin signaling, DUBs may also be dysregulated in AD. Among these enzymes, UCH-L1 has received particular attention because of its potential clinical relevance. Several clinical cohort studies have associated cerebrospinal fluid (CSF) UCH-L1 levels with disease progression [12,13], MMSE scores [14], and tau pathology [15]. Plasma UCH-L1 was also elevated in individuals with MCI due to AD and decreased following granulocyte-macrophage colony-stimulating factor (GM-CSF) treatment in patients with AD [16]. Further studies are needed to determine whether changes in UCH-L1 are directly involved in AD pathogenesis or primarily reflect ongoing neuronal injury.

Genetic evidence also links alterations in DUBs to AD. The Y allele and YY genotype of the UCH-L1 S18Y polymorphism have been reported to confer a protective effect against sporadic AD in women [17]. In addition, a cohort study of Chinese patients with AD and FTD identified four rare CYLD variants in approximately 1% of patients with AD, including three previously unreported variants, suggesting a potential association between rare CYLD variants and AD susceptibility [18]. Analysis of data from the Alzheimer’s Disease Neuroimaging Initiative (ADNI) further identified associations between USP10 genetic variants, hippocampal subfield volumes, and cognitive performance. Homozygous and heterozygous USP10 variants differed in the volume of the granule cell and molecular layer of the dentate gyrus (GC-ML-DG), a hippocampal subregion that is reduced in individuals with mild cognitive impairment.

Changes in DUB abundance have also been observed directly in the AD brain. USP10 [19], USP11 [20], and USP13 [21] are all reported to be elevated in postmortem brain tissues from patients with AD. Together, these findings indicate that DUB alterations in AD occur at multiple levels, including genetic variation, changes in protein abundance, and altered CSF levels, supporting further investigation into their potential roles in AD pathogenesis.

## 3. DUBs in Core Pathological Mechanisms of AD

### 3.1. DUBs Regulate Tau Clearance and Aggregation

Tau is a microtubule-associated protein that stabilizes microtubules and supports neuronal morphology and the axonal transport of organelles and other cellular cargoes in healthy neurons [22]. In AD, however, tau undergoes aberrant post-translational modifications that promote its dissociation from microtubules and subsequent aggregation into neurofibrillary tangles (NFTs) [23]. The resulting disruption of microtubule integrity and intracellular transport contributes to neuronal dysfunction and ultimately neuronal death [24]. DUBs regulate tau degradation and aggregation through multiple mechanisms, including direct deubiquitination of tau as well as indirect modulation of tau expression and phosphorylation.

#### 3.1.1. OTUB1

OTUB1 is an OTU-family deubiquitinase implicated in the regulation of tau pathology [25,26]. It directly interacts with tau and removes K48-linked polyubiquitin chains in a catalytic activity-dependent manner, thereby impairing tau degradation [26]. In primary neurons, Otub1 overexpression increased total tau and tau phosphorylation at Ser202/Thr205, as detected by the AT8 antibody, and promoted tau oligomerization and tau-seeded aggregation [26]. Consistent with these in vitro findings, Otub1 overexpression in the brains of TauP301S mice also increased AT8-positive and oligomeric tau [26]. Collectively, these findings indicate that OTUB1 promotes tau accumulation and aggregation in both in vitro and in vivo models, suggesting its potential as a therapeutic target for tau pathology. However, its relevance to human AD and the therapeutic effects of OTUB1 inhibition remain to be established.

#### 3.1.2. USP7

USP7 is a USP-family deubiquitinase that inhibits tau degradation and promotes its pathological accumulation. USP7 directly interacts with tau and counteracts CHIP-mediated tau ubiquitination, thereby increasing tau stability [27]. Consistent with this mechanism, USP7 overexpression increased cellular tau levels, whereas genetic depletion or pharmacological inhibition of USP7 reduced tau abundance [27]. In PS19 mice, USP7 knockdown reduced total and phosphorylated tau, attenuated gliosis and tau seeding activity, and ameliorated memory deficits [27]. Moreover, in neuronal and hippocampal slice cultures derived from rTg4510 mice, USP7 inhibition reduced seeded tau aggregation and increased the polyubiquitination of residual tau inclusions without substantially altering soluble tau levels [28]. Collectively, these findings support a role for USP7 in promoting tau stabilization and pathological aggregation, although its therapeutic selectivity requires further investigation.

#### 3.1.3. USP10

USP10 has emerged as a multifaceted regulator of pathological tau accumulation. It directly deubiquitinates tau and delays its degradation, thereby increasing total and phosphorylated tau levels and promoting tau aggregation [19]. Notably, USP10 is upregulated in postmortem AD brain tissue and APP/PS1 mice, but not in P301S mice. Exposure to Aβ42 also induces USP10 expression in primary neurons, suggesting that its upregulation may be associated with Aβ pathology [19]. Genetic depletion or pharmacological inhibition of USP10 also reduces seeded tau aggregation without substantially affecting soluble tau levels [28]. Together, these findings suggest that USP10 may represent a potential link between Aβ-associated pathology and tau aggregation, although this relationship remains to be directly established. In addition, USP10 facilitates the formation of tau-positive stress granules in neuronal cells through a mechanism independent of its deubiquitinating activity and colocalizes with phosphorylated tau inclusions in AD brain tissue [29]. Collectively, the catalytic and stress-granule-associated functions of USP10 may independently contribute to pathological tau accumulation. However, the mechanistic evidence is derived primarily from cellular models, and whether these mechanisms regulate tau pathology in vivo remains unclear.

#### 3.1.4. USP11

USP11 is an X-linked deubiquitinase that escapes complete X-chromosome inactivation, resulting in higher brain expression in females than in males [30]. USP11 protein levels were elevated in the postmortem AD patients and showed a stronger association with tau pathology in women than in men [20]. Mechanistically, USP11 deubiquitinates tau and thereby facilitates its acetylation at K274 and K281, promoting pathological tau accumulation [20]. Consistent with this mechanism, genetic deletion of Usp11 preferentially reduced acetylated tau accumulation and tau pathology and improved spatial memory in female P301S mice [20]. Collectively, these findings suggest that USP11 may contribute to the greater vulnerability of women to tau pathology, although its role in human AD remains primarily supported by associative evidence.

#### 3.1.5. USP13

USP13 protein levels are approximately twofold higher in postmortem AD brains than in control brains [21]. In primary cortical neurons, USP13 knockdown increased 20S proteasome activity and reduced phosphorylated tau levels. In TgAPP mice, USP13 knockdown reduced amyloid levels, whereas in rTg4510 mice, it increased phosphorylated tau ubiquitination and clearance [21]. Collectively, these findings implicate USP13 in both amyloid and tau accumulation, potentially through the regulation of proteasomal and autophagic clearance pathways. However, whether these pathological improvements translate into cognitive or behavioral benefits remains unknown.

#### 3.1.6. USP9

USP9X and USP9Y are homologous genes located on the X and Y chromosomes, respectively [31]. USP9Y exhibits pronounced sex-specific expression in the adult human brain and is significantly downregulated across several brain regions in men with AD [32]. In zebrafish embryos, usp9 knockdown significantly reduced the expression of both mapta and maptb. Similarly, combined USP9X/USP9Y knockdown reduced MAPT expression in DU145 cells [32]. By integrating these findings with prior studies and network analyses, the authors proposed that USP9 may regulate tau phosphorylation through pathways involving MARK4 and SNCA/GSK3β and influence MAPT transcription through a putative SMAD4-BACH1 pathway [32]. However, these proposed mechanisms have not been directly validated in neuronal or AD models. In contrast, localized Usp9x knockdown in the dentate gyrus of wild-type mice increased tau phosphorylation rather than reducing it [33]. These divergent findings suggest that the effects of USP9 on tau may depend on the paralogue examined, experimental model, brain region, cell type, and disease context. Further studies are required to define the respective contributions of USP9X and USP9Y to tau pathology and their potential sex-specific effects in AD.

#### 3.1.7. USP14

USP14 is a proteasome-associated deubiquitinase that can delay substrate degradation by removing ubiquitin chains [34]. The selective USP14 inhibitor IU1-47 accelerated the degradation of wild-type tau, the P301L and P301S mutants, and the A152T variant in primary neurons, with tau K174 being required for this effect [35]. However, the effects of USP14 inhibition on tau are not consistent across experimental systems. IU1 reduced tau levels in rat cortical neurons without enhancing proteasome activity and also caused neurotoxicity, while genetic loss of Usp14 did not alter total tau levels and instead increased tau phosphorylation in mouse brains [36]. More recently, wogonoside-functionalized ultrasmall Cu_2−x_Se nanoparticles were reported to inhibit USP14, reduce phosphorylated tau accumulation and synaptic damage, and improve cognition in AD mice [37]. Collectively, these findings identify USP14 as a potential regulator of tau turnover, although the inconsistent effects of its inhibition and the selectivity and neurological safety of USP14-directed interventions require further investigation.

#### 3.1.8. UCH-L1

UCHL1 is a neuron-enriched member of the UCH family that may exert a neuroprotective role in AD. UCH-L1 colocalizes with NFTs in AD brains, and soluble UCH-L1 levels are inversely correlated with NFT burden, suggesting a potential association with tau pathology [38,39]. Pharmacological inhibition or knockdown of UCH-L1 reduced the microtubule-binding capacity of tau and increased its phosphorylation and abnormal aggregation in cultured cells [40]. Consistently, UCH-L1 knockdown increased tau phosphorylation at Ser396, whereas its overexpression produced the opposite effect [41]. UCH-L1 may also regulate tau clearance through aggresome formation. Under proteasomal impairment, UCH-L1 inhibition reduced K63-linked ubiquitination and HDAC6 activity, disrupted the interaction between HDAC6 and tau, and consequently impaired tau aggresome formation [42]. Together, these findings suggest that UCH-L1 contributes to tau homeostasis through multiple mechanisms, although further studies are needed to establish whether restoring its activity can alleviate tau pathology in vivo.

### 3.2. DUBs Regulate Aβ Production and Metabolism

Aβ is generated through the sequential cleavage of amyloid precursor protein (APP) by β-site APP-cleaving enzyme 1 (BACE1) and γ-secretase [43]. Extracellular accumulation of Aβ leads to amyloid plaques and contributes to neuroinflammation, oxidative stress, and synaptic dysfunction, ultimately promoting neuronal dysfunction and death [44]. Notably, there is currently limited evidence that DUBs directly target Aβ peptides. Instead, DUBs appear to regulate Aβ levels indirectly by modulating the stability and degradation of amyloid precursor protein (APP) and BACE1, thereby contributing to Aβ pathology.

#### 3.2.1. USP8

USP8 is an endosome-associated deubiquitinase that regulates the trafficking and lysosomal degradation of membrane proteins [45]. By removing ubiquitin from BACE1 at K501, USP8 limits its lysosomal targeting and favors its recycling, thereby stabilizing BACE1 and promoting Aβ production [46]. Consistently, USP8 depletion in H4 human neuroglioma cells increased BACE1 ubiquitination and lysosomal degradation, resulting in reduced BACE1-mediated APP cleavage and Aβ production [46]. These findings suggest that targeting USP8 may offer a potential strategy to reduce Aβ production by promoting BACE1 degradation. However, whether USP8 inhibition can alleviate amyloid pathology in animal models or human AD remains to be established.

#### 3.2.2. USP14

As discussed above, pharmacological inhibition of USP14 has been reported to enhance proteasomal activity and promote the clearance of neurotoxic tau species in some experimental models. Moreover, IU1 and its novel analogues AA10 and AA51 attenuated Aβ-mediated toxicity in cellular and Caenorhabditis elegans models of AD [47,48]. Although the direct engagement of USP14 and the target selectivity of AA10 and AA51 require further validation, these findings suggest that the effects of USP14 inhibition on AD-related protein pathology may extend beyond tau to Aβ-associated proteostasis and neurotoxicity.

#### 3.2.3. USP25

USP25 is a member of the USP family whose gene is located on chromosome 21 [49]. Increased USP25 gene dosage has been proposed to contribute to the link between DS and AD pathology [50]. Under acute endoplasmic reticulum stress in CHO cells, USP25 levels are reduced, coinciding with increased APP ubiquitination and proteasomal degradation and decreased Aβ production [51]. By contrast, evidence from AD mouse models indicates that USP25 promotes amyloid pathology. USP25 overexpression exacerbates amyloid deposition in 5×FAD mice, whereas genetic depletion or pharmacological inhibition of USP25 reduces cerebral Aβ levels and amyloid burden [50]. Mechanistically, USP25 deubiquitinates APP and BACE1 and limits their lysosomal degradation, thereby increasing their abundance and promoting amyloidogenic APP processing [50]. These findings suggest that USP25 links chromosome 21 gene dosage to Aβ pathology by regulating the turnover of APP and BACE1. The apparently different effects observed under acute ER stress and in chronic AD models may reflect stress duration-dependent regulation of APP turnover, although the underlying mechanisms remain to be determined.

#### 3.2.4. UCH-L1

In addition to its effects on tau, UCH-L1 may also exert a neuroprotective role in AD by modulating Aβ pathology. Reduced UCHL1 levels have been reported in postmortem brain tissue from patients with AD [52]. Current evidence indicates that UCHL1 influences Aβ pathology by regulating the turnover of both BACE1 and APP [53]. UCHL1 overexpression reduces BACE1 abundance and amyloidogenic APP processing, resulting in decreased levels of C99 and Aβ [53]. Although UCHL1 promotes BACE1 degradation, whether BACE1 is a direct substrate of UCHL1 remains unclear. Available evidence suggests that reduced UCHL1 activity impairs the lysosomal degradation of BACE1 and promotes its accumulation [54]. UCHL1 also increases the pool of free ubiquitin and promotes APP ubiquitination and lysosomal degradation, thereby reducing Aβ production [55]. Consistent with these findings, UCHL1 overexpression in AD mouse models reduced cerebral Aβ levels and amyloid plaque burden and improved learning and memory deficits, further supporting its protective role against Aβ pathology.

A pathological feedback loop may also exist between UCHL1 and Aβ. Exposure to Aβ_1–42_ suppresses UCHL1 activity through NF-κB signaling, leading to impaired lysosomal degradation and accumulation of BACE1, which may further enhance Aβ production [54]. Collectively, these findings suggest that UCHL1 protects against Aβ pathology and associated neurotoxicity by facilitating APP and BACE1 turnover and maintaining neuronal function. However, the causal relationship between reduced UCHL1 levels and AD pathology and the substrate-specific mechanisms underlying its protective effects remain to be established.

### 3.3. DUBs Regulate Autophagy in AD

In addition to the ubiquitin-proteasome system, the autophagy-lysosomal system is another major degradation pathway for clearing misfolded proteins, protein aggregates, and damaged organelles. Impaired autophagy flux contributes to proteostasis disruption in AD, which can impair the clearance of abnormal proteins and exacerbate cellular damage [56]. DUBs participate in multiple stages of autophagy by editing ubiquitin chains on core autophagy proteins and selective autophagy receptors. For example, USP1 removes K63-linked ubiquitin modifications from ULK1 and supports classical autophagy by maintaining its proper subcellular localization [57]. OTUB1 stabilizes the mTORC1 inhibitor DEPTOR and thereby promotes autophagy [58]. USP13 removes K48- and K63-linked ubiquitin chains from p62/SQSTM1 at K7, thereby enhancing p62 stability and oligomerization and promoting p62-mediated selective autophagy [59]. Conversely, USP8 preferentially removes K11-linked ubiquitin chains from p62 at K420, thereby suppressing its autophagic activity [60].

These studies indicate that DUBs do not uniformly promote or inhibit autophagy. Instead, their effects depend on the specific substrate, ubiquitin chain linkage, and modification site. Notably, these mechanisms have been characterized primarily in non-AD models, and their involvement in AD-related autophagic dysfunction remains to be determined.

#### 3.3.1. USP7

USP7 may support autophagy in the brain by maintaining the intracellular pool of free ubiquitin. USP7 mediates the processing of the ubiquitin precursor UBA52, a process facilitated by MLKL [61]. Loss of MLKL impairs UBA52 processing and reduces free ubiquitin levels, resulting in decreased K63-linked polyubiquitination and abundance of the autophagy regulators BECN1 and ULK1. USP7 depletion similarly reduces free ubiquitin levels and impairs autophagic flux [61]. In vivo, Mlkl deficiency caused autophagic impairment and cognitive dysfunction and exacerbated pathology in an AD mouse model [61]. These findings implicate the MLKL–USP7–UBA52 axis in AD-related autophagic dysfunction, although the direct contribution of USP7 to AD pathology remains to be established.

#### 3.3.2. USP9X

Beyond its effects on tau expression and phosphorylation described above, USP9X may also influence tau clearance through the autophagy pathway. USP9X is upregulated in the hippocampus of P301S tauopathy mice, accompanied by reduced Raptor ubiquitination [8]. Mechanistically, USP9X deubiquitinates and stabilizes Raptor, thereby activating mTORC1 and suppressing autophagy. Conversely, in vitro treatment with WP1130 increased Raptor ubiquitination and degradation, enhanced autophagy, and promoted the degradation of tau and phosphorylated tau [8]. However, USP9X knockdown in the dentate gyrus of wild-type mice was associated with autophagic dysfunction [33], suggesting that its effects on autophagy may depend on the disease state and brain region.

#### 3.3.3. USP11

USP11 has also been identified as a negative regulator of autophagy. In mammalian cells, USP11 knockout enhances autophagic flux by promoting the association of NRBF2 with PI3KC3-C1 and accelerating mTOR turnover [62]. Depletion of the USP11 homolog H34C03.2 in Caenorhabditis elegans similarly activates autophagy and alleviates Aβ_42_ aggregation-induced paralysis without significantly altering Aβ_42_ monomer levels [62]. These findings suggest that USP11 inhibition may alleviate Aβ-associated proteotoxicity by enhancing autophagy, although its relevance to the mammalian AD brain remains to be established.

#### 3.3.4. USP14

In addition to regulating tau turnover through its effects on proteasomal activity, USP14 also participates in the regulation of autophagy. The selective USP14 inhibitor IU1-47 was reported to enhance autophagic flux in primary neurons [35]. However, another study examining the crosstalk between the UPS and autophagy showed that proteasome activation induced by USP14 inhibition accelerated UVRAG degradation, thereby impairing autophagosome–lysosome fusion and reducing autophagic flux [63]. These apparently divergent findings suggest that the effects of USP14 inhibition on autophagy may depend on the experimental context. Together, these studies indicate that the effects of USP14 inhibition on tau clearance may reflect the balance between proteasomal degradation and autophagic flux.

### 3.4. DUBs Regulate Neuroinflammation

Neuroinflammation is an important component of AD pathogenesis and is largely mediated by microglia [64]. Microglia contribute to tissue homeostasis by clearing Aβ and cellular debris, whereas their persistent dysregulation can promote inflammatory responses, synaptic dysfunction, and neuronal injury [65]. Recent studies have increasingly implicated DUBs in the regulation of microglial responses in AD. By controlling the ubiquitination and stability of inflammatory regulators, several DUBs modulate microglial activation, inflammatory signaling, and related pathological responses.

#### 3.4.1. USP25

As discussed above, USP25 is encoded on chromosome 21, and its increased gene dosage may contribute to the development of DS-associated early-onset AD. In addition, USP25 may mediate trisomy 21-induced neuroinflammation. USP25 overexpression exacerbates microglial activation, neuroinflammation, synapse loss, and cognitive impairment, whereas genetic reduction of USP25 alleviates these abnormalities in 5×FAD mice [9]. Mechanistically, USP25 deficiency suppresses pro-inflammatory cytokine production and excessive microglial engulfment of synapses while partially restoring homeostatic microglial signatures [9]. These findings suggest that USP25 may connect trisomy 21 gene dosage with dysregulated microglial responses and synaptic injury in AD.

#### 3.4.2. OTUD1

OTUD1 is a member of the OTU family and is upregulated in AD mouse models and Aβ-stimulated BV2 microglial cells. Genetic depletion of OTUD1 attenuates neuroinflammation and cognitive impairment [66]. Mechanistically, OTUD1 removes K48-linked polyubiquitin chains from C/EBPβ at Lys253, thereby stabilizing C/EBPβ and enhancing C/EBPβ-mediated NF-κB signaling in microglia [66]. These findings identify the OTUD1–C/EBPβ axis as a potential regulator of AD-associated neuroinflammation, although its relevance to human microglia remains to be determined.

#### 3.4.3. OTUD6A

OTUD6A is another member of the OTU family and is upregulated in microglia across multiple AD mouse models. Genetic deletion of Otud6a attenuates neuroinflammation and cognitive deficits [67]. Similarly to OTUD1, OTUD6A removes K48-linked polyubiquitin chains from C/EBPβ at Lys253, thereby stabilizing C/EBPβ and enhancing NF-κB signaling and pro-inflammatory cytokine production [67]. The convergence of OTUD1 and OTUD6A on the same substrate and ubiquitination site suggests that C/EBPβ may represent a common inflammatory regulatory node for OTU-family DUBs. However, whether these enzymes function redundantly or exert context-specific effects remains unclear.

#### 3.4.4. YOD1

YOD1 is also a member of the OTU family and is upregulated in Aβ-stimulated microglia and AD mouse models. Microglial depletion of YOD1 restores microglial migration and phagocytosis, attenuates inflammatory responses, and ameliorates cognitive deficits in AD mice [68]. Mechanistically, YOD1 removes K48-linked polyubiquitin chains from MYH9 and maintains its stability, thereby modulating microglial homeostasis [68]. Unlike OTUD1 and OTUD6A, which converge on C/EBPβ, YOD1 regulates microglial function through MYH9, indicating that OTU-family DUBs modulate AD-related neuroinflammation through distinct molecular pathways.

#### 3.4.5. USP53

USP53 expression is elevated in postmortem brain tissue from patients with AD. In 5×FAD mice, genetic deletion of Usp53 attenuates microglial and astrocytic reactivity, reduces pro-inflammatory cytokine production and tau accumulation, and improves learning and memory [69]. Mechanistically, USP53 interacts with and deubiquitinates NOTCH2, thereby stabilizing NOTCH2 and enhancing IKKβ/NF-κB signaling in microglia. Consistently, USP53 knockdown suppresses Aβ-induced inflammatory responses in cultured microglia [69]. These findings suggest that USP53 may promote AD-associated neuroinflammation by stabilizing NOTCH2 and enhancing downstream NF-κB signaling.

### 3.5. DUBs Regulate Mitochondrial Dysfunction

Mitochondrial dysfunction is an important pathological feature of Alzheimer’s disease. Neurons rely heavily on mitochondria to maintain energy production, calcium homeostasis, and synaptic function. In AD, impaired oxidative phosphorylation, excessive production of reactive oxygen species (ROS), altered mitochondrial dynamics, and defective mitophagy are widely observed [70]. These abnormalities not only directly compromise neuronal function but also interact with Aβ pathology, tau pathology, and neuroinflammation, collectively contributing to disease progression [71].

Studies conducted predominantly in Parkinson’s disease-related or other non-AD models indicate that DUBs regulate mitochondrial quality control by modifying Parkin or its mitochondrial substrates. USP8 removes K6-linked ubiquitin chains from Parkin, thereby promoting its recruitment to depolarized mitochondria and supporting Parkin-dependent mitophagy [72]. However, USP8 downregulation has also been shown to enhance Parkin-independent basal mitophagy, suggesting that its effects are dependent on the mitophagy pathway and cellular context [73]. Genetic or pharmacological inhibition of USP14 similarly promotes mitophagy through a DRP1-dependent and MFN2-dependent mechanism that is independent of the canonical PINK1/Parkin pathway [74,75]. By contrast, USP15 and USP35 inhibit Parkin-mediated mitophagy without markedly affecting Parkin recruitment to damaged mitochondria [76,77]. USP33 directly deubiquitinates Parkin, and its deficiency enhances Parkin ubiquitination and mitochondrial recruitment, thereby promoting mitophagy [78]. Together, these findings demonstrate that DUBs regulate mitophagy at multiple levels by targeting Parkin or its mitochondrial substrates. Although direct evidence linking these DUBs to mitochondrial dysfunction in AD remains limited, findings from other experimental systems provide mechanistic clues and identify potential directions for future investigation in AD.

#### 3.5.1. USP19

USP19 has been implicated in AD-associated mitochondrial dysfunction through its regulation of oxidative stress, ferroptosis, and mitochondrial dynamics [79]. In Aβ_1–40_-treated SH-SY5Y cells, USP19 knockdown restored MFN1 and MFN2 expression, reduced DRP1 levels, and attenuated iron accumulation, oxidative stress, and ferroptosis-associated cell injury [79]. Mechanistically, USP19 interacts with and stabilizes NOX4 by limiting its ubiquitination, while NOX4 re-expression partially reverses the mitochondrial and cellular protective effects of USP19 knockdown [79]. These findings suggest that USP19 may exacerbate mitochondrial dysfunction by stabilizing NOX4 and amplifying oxidative stress. However, the evidence is currently limited to an Aβ-induced cellular model, and the relevance of this mechanism to AD animal models and human brain tissue remains to be established.

#### 3.5.2. USP30

USP30 is an outer mitochondrial membrane-localized DUB that negatively regulates PINK1/Parkin-dependent mitophagy by removing ubiquitin chains from mitochondrial surface proteins. Accordingly, USP30 inhibition enhances mitochondrial ubiquitination and facilitates the clearance of damaged mitochondria [80]. Pharmacological inhibition of USP30 has also emerged as a potential strategy for improving mitochondrial quality control. Among the reported USP30 inhibitors, CMPD-39 has been shown to enhance mitophagy [81], whereas MTX325 has demonstrated neuroprotective effects in a mouse model of Parkinson’s disease [82]. However, the therapeutic potential of these inhibitors in AD remains to be established.

In AD-related models, miR-137-5p was reported to target USP30 and attenuate Aβ_1–42_-induced neurotoxicity in SH-SY5Y cells, whereas its administration in mice improved cognitive function and reduced Aβ deposition, tau hyperphosphorylation, and neuronal apoptosis [83]. However, these findings do not establish that the observed protective effects are mediated specifically by USP30-dependent mitophagy, because miR-137-5p may regulate multiple targets. Further genetic rescue and in vivo target-validation studies are therefore required to determine whether USP30 directly links impaired mitochondrial quality control to AD pathology.

#### 3.5.3. UCH-L1

UCH-L1 may also contribute to tau-associated dysregulation of mitochondrial quality control. In primary neurons, N-terminally truncated tau (NH_2_htau) induces excessive Parkin-dependent mitophagy accompanied by aberrant recruitment of UCH-L1 to mitochondria [84]. Genetic depletion of either UCH-L1 or Parkin suppresses excessive mitochondrial clearance, restores mitochondrial and synaptic content, and partially protects neurons against NH_2_htau-induced death [84]. Moreover, endogenous NH_2_htau was found to be stably associated with Parkin and UCH-L1 in synaptic mitochondria isolated from patients with AD, supporting the potential disease relevance of this mechanism [84]. These findings suggest that UCH-L1 contributes to detrimental mitochondrial clearance in the presence of NH_2_htau. However, whether this effect depends on its deubiquitinating activity and which mitochondrial substrates are involved remain unclear.

### 3.6. DUBs Regulate Other AD-Related Pathological Processes

Beyond the pathological processes discussed above, DUBs have also been implicated in other aspects of AD pathophysiology, including synaptic dysfunction and impaired neural progenitor cell function. USP46 regulates the ubiquitination and turnover of AMPA receptors (AMPARs). In primary neurons, Aβ exposure reduces USP46 expression while increasing the E3 ligase NEDD4, changes associated with enhanced AMPAR ubiquitination and degradation. Similar alterations have also been observed in brain tissue from patients with AD [85]. UCH-L1 also supports synaptic plasticity by maintaining neuronal ubiquitin homeostasis and PKA–CREB signaling. Restoration of UCHL1 activity alleviates Aβ-induced long-term potentiation deficits and improves contextual memory in APP/PS1 mice [86]. Beyond synaptic regulation, USP16 may impair neural regeneration by restricting neural progenitor cell self-renewal. In Tg-SwDI mice and APP-mutant human cell models, USP16 depletion restores progenitor cell function and ameliorates cognitive deficits, potentially through modulation of H2AK119 monoubiquitination and CDKN2A/BMP signaling [87].

Overall, DUBs have been implicated in a range of AD-related pathological processes through the regulation of different substrates and signaling pathways. The reported roles of individual DUBs and the corresponding experimental evidence are summarized in Table 1.

## 4. DUBs Involved in Multiple AD-Related Pathological Processes

**Figure 2:**
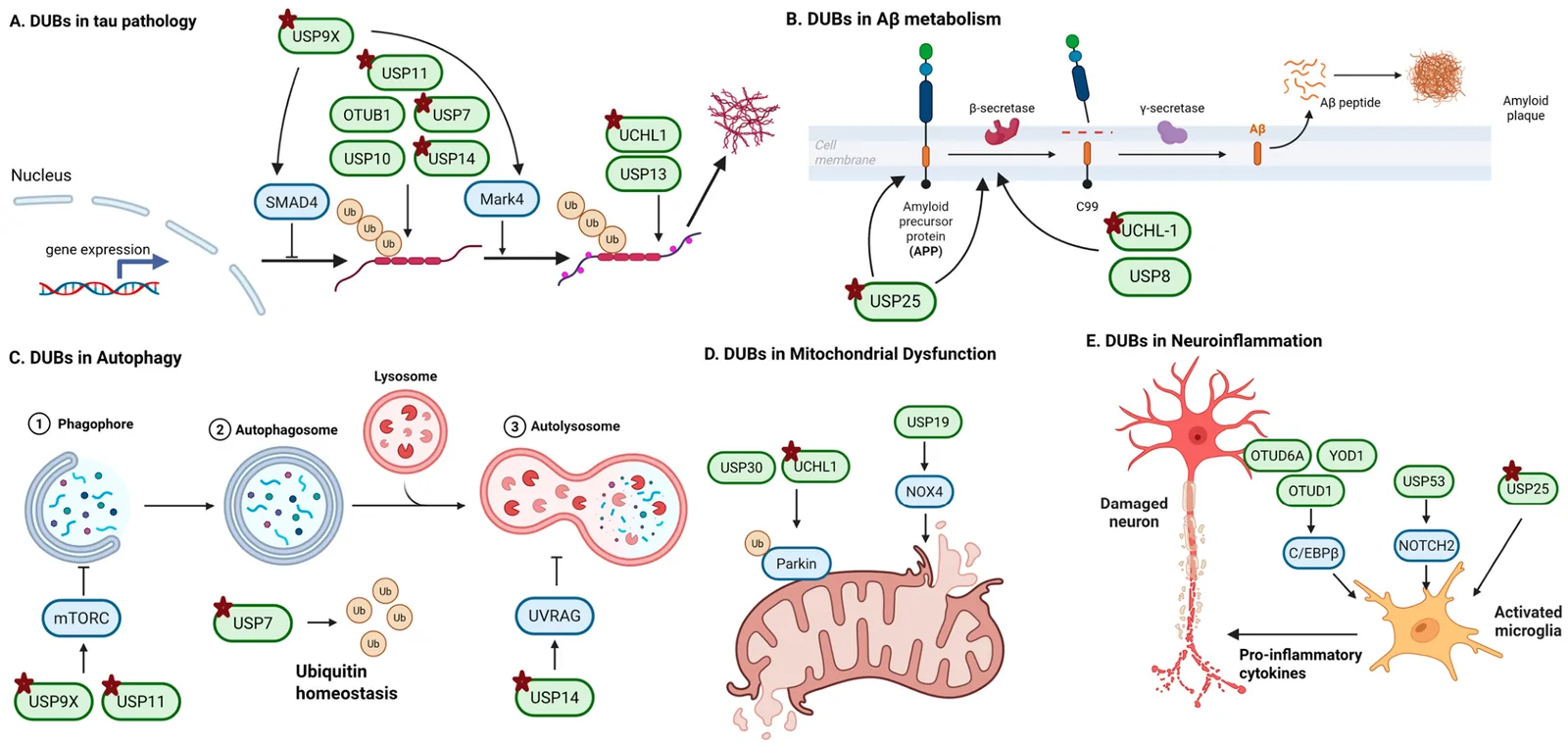
Roles of deubiquitinating enzymes in major pathological processes associated with Alzheimer’s disease. DUBs participate in multiple aspects of AD pathology through the regulation of different substrates and signaling pathways. (**A**) DUBs regulate tau homeostasis by modulating tau expression, phosphorylation, ubiquitination, and degradation, thereby influencing tau accumulation and aggregation. (**B**) DUBs affect Aβ metabolism by regulating proteins involved in amyloid precursor protein (APP) processing and Aβ production. (**C**) DUBs modulate different stages of the autophagy–lysosome pathway, including autophagy initiation, autophagosome formation, and lysosomal degradation. (**D**) DUBs contribute to mitochondrial dysfunction by regulating mitochondrial quality-control pathways and oxidative stress. (**E**) DUBs regulate neuroinflammatory responses, particularly microglial activation and inflammatory signaling. A star indicates DUBs implicated in multiple AD-related pathological processes. (See Figure 2 on the next page.)

Taken together, the available evidence indicates that an individual DUB may influence multiple pathological processes in AD by acting on distinct substrates and signaling pathways. Figure 2 summarizes the involvement of DUBs in different AD-related pathological processes, with those implicated in multiple processes marked by asterisks. For example, USP25 promotes Aβ production by stabilizing APP and BACE1 while also exacerbating microglia-mediated neuroinflammation. These effects may act in concert to aggravate AD pathology [9,50]. USP7 stabilizes tau and promotes its pathological accumulation but also contributes to the maintenance of intracellular ubiquitin homeostasis and autophagic function, suggesting that its different activities may have distinct or even opposing pathological consequences [27,61]. USP9X exhibits similarly complex and context-dependent effects. In addition to its potential regulation of tau expression and phosphorylation, elevated USP9X stabilizes Raptor and activates mTORC1 in P301S mice, thereby suppressing autophagic tau clearance. By contrast, Usp9x knockdown in the dentate gyrus of wild-type mice results in autophagic dysfunction [8,32,33]. Together, these examples highlight the potential connections among different AD-related pathological processes regulated by DUBs. USP25 may contribute to both amyloid pathology and neuroinflammation, whereas USP7 may exert opposing effects on tau accumulation and autophagic function. USP9X-mediated regulation of autophagy further provides a mechanistic link to tau clearance. These findings suggest that the overall effects of a DUB cannot be inferred from a single substrate or downstream phenotype but depend on its substrate repertoire and the specific cellular and pathological context, including cell type, brain region, and disease stage.

In addition to their mechanistic actions on multiple substrates and pathways, interventions targeting certain DUBs have produced improvements across several AD-related pathological phenotypes. For example, the USP11-targeting compound IsoLiPro reduced tau and amyloid pathologies, attenuated synaptic damage, and improved cognitive function in 3×Tg-AD and 5×FAD mice [88]. Genetic deletion of Usp53 similarly attenuated neuroinflammation and tau accumulation and improved learning and memory in 5×FAD mice [69]. However, such broad phenotypic improvements do not necessarily demonstrate that these DUBs directly regulate multiple pathological pathways. Beyond the substrates and signaling pathways that have been identified, the remaining pathological changes may represent direct effects, secondary downstream consequences, or off-target effects of the experimental interventions. The causal relationships among these effects therefore remain to be established.

Importantly, the different functions of individual DUBs have generally been identified in separate experimental models, and few studies have simultaneously examined their effects on multiple pathological processes within the same system. Current evidence therefore supports describing some DUBs as multifaceted regulators of AD pathology but remains insufficient to establish that they directly coordinate interactions among distinct pathological processes. Future studies combining cell-type-specific manipulation, longitudinal analysis across disease stages, and integrated proteomic and ubiquitinomic profiling will help determine whether these DUBs represent key regulatory nodes within the AD pathological network. Their broad substrate repertoires and context-dependent functions also suggest that targeting DUBs may affect multiple pathological components simultaneously. However, their therapeutic value must be evaluated in relation to target selectivity and the preservation of their physiological functions.

## 5. Pharmacological Targeting of DUBs in AD

DUBs have well-defined catalytic activity and structurally accessible catalytic pockets, making them attractive targets for pharmacological intervention within the ubiquitin system [89]. Rather than broadly interfering with protein degradation, targeting individual DUBs may offer a more selective approach to modulating disease-associated ubiquitin signaling, given their distinct substrate and ubiquitin-chain preferences [7]. The involvement of DUBs in multiple AD-related pathological processes provides a rationale for exploring DUBs as potential therapeutic targets in AD.

Several studies have provided initial pharmacological evidence supporting this possibility. For example, administration of the USP25 inhibitor AZ1 reduced amyloid plaque burden in brain sections from 5×FAD mice [50]. The USP14 inhibitor IU1 and its more potent derivative IU1-47 have also been investigated for their ability to enhance proteasomal degradation. In particular, IU1-47 reduced total and phosphorylated tau levels in primary cortical neurons derived from 3×FAD mice and in human iPSC-derived neurons [35].

More recently, additional therapeutic approaches have been explored. IsoLiPro, a compound targeting USP11, increased tau ubiquitination and promoted tau clearance. Long-term oral administration of IsoLiPro reduced total and phosphorylated tau levels in AD transgenic mice. Treatment also attenuated amyloid deposition and synaptic damage and improved cognitive performance [88]. In addition, wogonoside-functionalized ultrasmall Cu_2−x_Se nanoparticles (CSPW NPs) have been developed to inhibit USP14 and enhance ubiquitin–proteasome-mediated clearance of hyperphosphorylated tau. With cell membrane coating and focused ultrasound assistance, these nanoparticles can more efficiently cross the blood–brain barrier and target neurons. In AD mice, this strategy reduced tau pathology and synaptic loss, improved microtubule stability and synaptic integrity, and alleviated cognitive deficits [37]. Together, these studies demonstrate that DUBs are pharmacologically tractable and support the therapeutic potential of targeting DUBs in AD. However, most of the available evidence remains limited to cellular and animal models, and clinical translation is still at an early stage.

Important challenges need to be addressed before DUB-targeted therapies can be considered for clinical application. First, achieving sufficient selectivity remains a major challenge [89]. Most DUBs are cysteine proteases, and members of the same family often share conserved catalytic features. Compounds directed against the catalytic site may therefore inhibit related DUBs or other cysteine-dependent enzymes. Structure-guided drug design and the identification of allosteric sites outside the conserved catalytic center may offer ways to improve target selectivity [90]. Second, drug accessibility is particularly important for AD. Blood–brain barrier permeability, brain exposure, and pharmacokinetic properties require careful evaluation [91]. Many existing DUB inhibitors were not originally developed for neurodegenerative diseases, and their pharmacological properties in the central nervous system remain poorly characterized [92]. Third, the broad biological functions of DUBs add another layer of complexity. Individual DUBs often act on multiple substrates and may participate in distinct or even opposing cellular processes. Their ability to influence several AD-related pathways may offer therapeutic advantages, but may also increase the risk of unintended effects. A better understanding of the overall consequences of modulating individual DUBs in the AD brain will therefore be important for evaluating their therapeutic potential.

Importantly, therapeutic modulation of DUBs should not be limited to inhibition. Some DUBs appear to have protective functions in AD. For example, loss of UCH-L1 function has been associated with increased tau phosphorylation, impaired proteostasis, and synaptic dysfunction [55]. In such cases, restoring DUB expression or enhancing its activity may be more appropriate than inhibition. Future therapeutic strategies should therefore be tailored to the specific role of each DUB, with inhibition, activation, or functional restoration considered according to its effects in AD. Improving substrate selectivity may provide an additional means of limiting interference with the physiological functions of DUBs.

## 6. Conclusions

Alzheimer’s disease is a neurodegenerative disorder involving multiple pathological processes. Research has traditionally focused on the accumulation of abnormal proteins, particularly Aβ and tau. However, impaired autophagy, mitochondrial dysfunction, neuroinflammation, and synaptic damage are increasingly recognized as important components of AD pathogenesis, with some of these alterations emerging at early stages of the disease. DUBs are important regulators of cellular proteostasis, and accumulating evidence links their dysregulation not only to Aβ and tau metabolism but also to autophagy, mitochondrial function, neuroinflammation, and synaptic function. In this review, we summarize the roles of DUBs in these AD-related pathological processes.

Despite recent progress, substantial gaps remain in our understanding of DUBs in AD. Only a limited number of DUBs have been investigated in AD-related models, while many mechanisms involving toxic protein clearance and mitochondrial quality control have been characterized primarily in Parkinson’s disease or other neurodegenerative diseases. Identifying additional AD-associated DUBs and establishing their causal relevance will therefore be important. For DUBs that affect multiple pathological processes, identifying their direct substrates and downstream pathways will be particularly important for distinguishing direct effects from secondary changes. Further evidence from human brain tissue, patient-derived cells, and brain organoids is also needed, as changes in DUB abundance alone may not reflect changes in catalytic activity or substrate regulation. Finally, although pharmacological modulation of several DUBs has shown beneficial effects in experimental models, therapeutic studies remain largely preclinical. Further work is needed to establish appropriate targets and determine whether individual DUBs should be inhibited or restored, while addressing drug selectivity, brain exposure, and long-term safety.

## Figures and Tables

**Table 1:** Deubiquitinating enzymes involved in major AD-related pathological processes.

AD-Related Process	DUB	Reported Alteration	Mechanism	Ref
Tau pathology	OTUB1	-	Removes K48-linked polyubiquitin chains from tau and inhibits tau degradation.	[26]
	USP7	-	Counteracts CHIP-mediated tau polyubiquitination and stabilizes tau.	[27]
	USP10	Upregulated in human AD and APP/PS1 mice	Deubiquitinates tau and slows its turnover and promotes tau-positive stress granule formation, facilitating tau aggregation.	[19,29]
	USP11	Upregulated in human AD and P301S mice	Deubiquitinates tau and facilitates acetylation at K274 and K281, thereby promoting pathological tau accumulation.	[20]
	USP13	Upregulated in human AD	Restrains tau ubiquitination and clearance, thereby promoting phosphorylated tau accumulation.	[21]
	USP9	-	Modulates tau phosphorylation and regulates MAPT expression.	[32]
	USP14	-	Restrains tau degradation.	[35]
Aβ pathology	USP8	-	Deubiquitinates BACE1 at K501, stabilizing BACE1 and promoting Aβ production.	[46]
	USP14	-	Limits proteasomal clearance of aggregation-prone proteins, contributing to Aβ/APP-C99-mediated proteotoxicity.	[48]
	USP25	-	Reduces ubiquitination and lysosomal degradation of APP and BACE1, promoting APP β-cleavage and Aβ generation.	[50]
	UCH-L1	Downregulated in human AD and APP/PS1 mice	Promotes APP degradation and reduces BACE1 stability, thereby limiting APP processing products and Aβ production.	[53]
Autophagy	USP7	-	Processes UBA52 to maintain the free ubiquitin pool, thereby preserving K63-linked ubiquitination and stability of BECN1 and ULK1 and supporting autophagic flux.	[61]
	USP9X	Upregulated in P301S mice	Deubiquitinates and stabilizes Raptor, activates mTORC1, and impairs autophagic flux, thereby limiting tau clearance.	[8]
	USP11	-	Restricts autophagy by limiting class III PI3K complex I activity.	[62]
	USP14		Restrains proteasomal activity and may limit autophagic clearance in a context-dependent manner.	[63]
Neuroinflammation	USP25	-	Promotes microglial activation and inflammatory responses	[9]
	OTUD1	Upregulated in multiple AD mouse models	Deubiquitinates and stabilizes C/EBPβ, thereby enhancing microglial inflammatory signaling.	[66]
	OTUD6A	Upregulated in multiple AD mouse models	Deubiquitinates and stabilizes C/EBPβ, thereby enhancing microglial inflammatory signaling.	[67]
	YOD1	Upregulated in multiple AD mouse models	Deubiquitinates and stabilizes MYH9, thereby altering microglial homeostasis.	[68]
	USP53	Upregulated in human AD	Deubiquitinates and stabilizes NOTCH2, promoting IKKβ/NF-κB signaling and microglial neuroinflammation.	[69]
Mitochondrial dysfunction	USP19	-	Promotes NOX4-associated mitochondrial injury and ferroptotic changes in response to Aβ.	[79]
	USP30	-	Opposes mitochondrial ubiquitination and mitophagy.	[80,83]
	UCH-L1	-	Contributes to NH_2_htau-induced excessive mitophagy.	[84]
Other	USP46	Downregulated in human AD	Deubiquitinates AMPARs and regulates synaptic receptor stability.	[85]
	UCH-L1	Downregulated	Supports synaptic function.	[86]
	USP16	-	USP16 reduction restores NPC function and improves cognitive phenotypes.	[87]

## Data Availability

No new data were created or analyzed in this study. Data sharing is not applicable to this article.

## Abbreviations

The following abbreviations are used in this manuscript:

AD	Alzheimer’s disease
Aβ	Amyloid-β
APP	Amyloid precursor protein
AMPAR	α-Amino-3-hydroxy-5-methyl-4-isoxazolepropionic acid receptor
BACE1	β-Site APP-cleaving enzyme 1
CSF	Cerebrospinal fluid
DUB	Deubiquitinating enzyme
DS	Down syndrome
FTD	Frontotemporal dementia
MCI	Mild cognitive impairment
MMSE	Mini-Mental State Examination
NFT	Neurofibrillary tangle
OTU	Ovarian tumor protease
ROS	Reactive oxygen species
UCH	Ubiquitin C-terminal hydrolase
UPS	Ubiquitin–proteasome system
USP	Ubiquitin-specific protease

## References

[1] Apostolova L.G. (2016). Alzheimer Disease. Continuum.

[2] Angst G., Jia N., Esqueda L.E.T., Fan Y., Cai Q., Wang C. (2025). Autophagy in alzheimer disease pathogenesis and its therapeutic values. Autophagy Rep..

[3] Cozachenco D., Ribeiro F.C., Ferreira S.T. (2023). Defective proteostasis in Alzheimer’s disease. Ageing Res. Rev..

[4] Liu B., Ruan J., Chen M., Li Z., Manjengwa G., Schlüter D., Song W., Wang X. (2022). Deubiquitinating enzymes (DUBs): Decipher underlying basis of neurodegenerative diseases. Mol. Psychiatry.

[5] Akutsu M., Dikic I., Bremm A. (2016). Ubiquitin chain diversity at a glance. J. Cell Sci..

[6] Wu Y., Deng Y., Ai Q., Li Y., Qin F., Hammad M., Meng Z., Xu X., Wei J., Yu H. (2025). Deubiquitinating enzymes in parkinson’s disease: Molecular mechanisms and therapeutic potential. Mol. Med..

[7] Clague M.J., Urbé S., Komander D. (2019). Breaking the chains: Deubiquitylating enzyme specificity begets function. Nat. Rev. Mol. Cell Biol..

[8] Zheng S., Zhu J., Wang C., Wu Y., Sun S., Guo H., Chang Y., Ma R., Li G. (2024). USP9X-mediated deubiquitination of Raptor contributes to autophagy impairment and memory deficits in P301S mice. Cell Commun. Signal..

[9] Zheng Q., Li G., Wang S., Zhou Y., Liu K., Gao Y., Zhou Y., Zheng L., Zhu L., Deng Q. (2021). Trisomy 21-induced dysregulation of microglial homeostasis in Alzheimer’s brains is mediated by USP25. Sci. Adv..

[10] Qin B., Chen X., Wang F., Wang Y. (2024). DUBs in Alzheimer’s disease: Mechanisms and therapeutic implications. Cell Death Discov..

[11] Jia F., Fu L. (2025). Roles of Ubiquitin Ligases and Deubiquitylases in Alzheimer’s Disease. Mol. Neurobiol..

[12] Paciotti S., Wojdała A.L., Bellomo G., Toja A., Chipi E., Piersma S.R., Pham T.V., Gaetani L., Jimenez C.R., Parnetti L. (2023). Potential diagnostic value of CSF metabolism-related proteins across the Alzheimer’s disease continuum. Alzheimer’s Res. Ther..

[13] Öhrfelt A., Johansson P., Wallin A., Andreasson U., Zetterberg H., Blennow K., Svensson J. (2016). Increased Cerebrospinal Fluid Levels of Ubiquitin Carboxyl-Terminal Hydrolase L1 in Patients with Alzheimer’s Disease. Dement. Geriatr. Cogn. Disord. Extra.

[14] Schulz I., Kruse N., Gera R.G., Kremer T., Cedarbaum J., Barbour R., Zago W., Schade S., Otte B., Bartl M. (2021). Systematic Assessment of 10 Biomarker Candidates Focusing on α-Synuclein-Related Disorders. Mov. Disord..

[15] Wojdała A.L., Bellomo G., Gaetani L., Toja A., Chipi E., Shan D., Chiasserini D., Parnetti L. (2023). Trajectories of CSF and plasma biomarkers across Alzheimer’s disease continuum: Disease staging by NF-L, p-tau181, and GFAP. Neurobiol. Dis..

[16] Sillau S.H., Coughlan C., Ahmed M.M., Nair K., Araya P., Galbraith M.D., Ritchie A., Wang A.C.-J., Elos M.T., Bettcher B.M. (2026). Blood measure of neuronal death is exponentially higher with age, especially in females, and halted in Alzheimer’s disease by GM-CSF treatment. Cell Rep. Med..

[17] Xue S., Jia J. (2006). Genetic association between Ubiquitin Carboxy-terminal Hydrolase-L1 gene S18Y polymorphism and sporadic Alzheimer’s disease in a Chinese Han population. Brain Res..

[18] Xiao X., Xu T., Liu H., Liu X., Liao X., Zhou Y., Zhou L., Wang X., Zhu Y., Yang Q. (2022). CYLD variants identified in Alzheimer’s disease and frontotemporal dementia patients. Ann. Clin. Transl. Neurol..

[19] Wei Z., Zeng K., Hu J., Li X., Huang F., Zhang B., Wang J.Z., Liu R., Li H.L., Wang X. (2022). USP10 deubiquitinates Tau, mediating its aggregation. Cell Death Dis..

[20] Yan Y., Wang X., Chaput D., Shin M.K., Koh Y., Gan L., Pieper A.A., Woo J.A., Kang D.E. (2022). X-linked ubiquitin-specific peptidase 11 increases tauopathy vulnerability in women. Cell.

[21] Liu X., Hebron M.L., Mulki S., Wang C., Lekah E., Ferrante D., Shi W., Kurd-Misto B., Moussa C. (2019). Ubiquitin Specific Protease 13 Regulates Tau Accumulation and Clearance in Models of Alzheimer’s Disease. J. Alzheimers Dis..

[22] Avila J., Lucas J.J., Perez M., Hernandez F. (2004). Role of tau protein in both physiological and pathological conditions. Physiol. Rev..

[23] Jiang G., Xie G., Li X., Xiong J. (2025). Cytoskeletal Proteins and Alzheimer’s Disease Pathogenesis: Focusing on the Interplay with Tau Pathology. Biomolecules.

[24] Tiwari S., Atluri V., Kaushik A., Yndart A., Nair M. (2019). Alzheimer’s disease: Pathogenesis, diagnostics, and therapeutics. Int. J. Nanomed..

[25] Balakirev M.Y., Tcherniuk S.O., Jaquinod M., Chroboczek J. (2003). Otubains: A new family of cysteine proteases in the ubiquitin pathway. EMBO Rep..

[26] Wang P., Joberty G., Buist A., Vanoosthuyse A., Stancu I.C., Vasconcelos B., Pierrot N., Faelth-Savitski M., Kienlen-Campard P., Octave J.N. (2017). Tau interactome mapping based identification of Otub1 as Tau deubiquitinase involved in accumulation of pathological Tau forms in vitro and in vivo. Acta Neuropathol..

[27] Kim J., de Haro M., Al-Ramahi I., Garaicoechea L.L., Jeong H.H., Sonn J.Y., Tadros B., Liu Z., Botas J., Zoghbi H.Y. (2023). Evolutionarily conserved regulators of tau identify targets for new therapies. Neuron.

[28] Volbracht C., Fog K. (2025). Deubiquitinating Enzymes Ubiquitin-Specific Proteases 7 and 10 Regulate TAU Aggregation. Int. J. Mol. Sci..

[29] Piatnitskaia S., Takahashi M., Kitaura H., Katsuragi Y., Kakihana T., Zhang L., Kakita A., Iwakura Y., Nawa H., Miura T. (2019). USP10 is a critical factor for Tau-positive stress granule formation in neuronal cells. Sci. Rep..

[30] Tukiainen T., Villani A.C., Yen A., Rivas M.A., Marshall J.L., Satija R., Aguirre M., Gauthier L., Fleharty M., Kirby A. (2017). Landscape of X chromosome inactivation across human tissues. Nature.

[31] Jones M.H., Furlong R.A., Burkin H., Chalmers I.J., Brown G.M., Khwaja O., Affara N.A. (1996). The Drosophila developmental gene fat facets has a human homologue in Xp11.4 which escapes X-inactivation and has related sequences on Yq11.2. Hum. Mol. Genet..

[32] Köglsberger S., Cordero-Maldonado M.L., Antony P., Forster J.I., Garcia P., Buttini M., Crawford A., Glaab E. (2017). Gender-Specific Expression of Ubiquitin-Specific Peptidase 9 Modulates Tau Expression and Phosphorylation: Possible Implications for Tauopathies. Mol. Neurobiol..

[33] Qi X., Ying M., Wang A., Shi K., Zhong G., Lu Y., Liu C., Guo Y. (2025). Downregulation of USP9X in the DG Region of the Hippocampus Leads to AD-Like Cognitive Dysfunction in Mice. CNS Neurosci. Ther..

[34] Lee B.H., Lee M.J., Park S., Oh D.C., Elsasser S., Chen P.C., Gartner C., Dimova N., Hanna J., Gygi S.P. (2010). Enhancement of proteasome activity by a small-molecule inhibitor of USP14. Nature.

[35] Boselli M., Lee B.H., Robert J., Prado M.A., Min S.W., Cheng C., Silva M.C., Seong C., Elsasser S., Hatle K.M. (2017). An inhibitor of the proteasomal deubiquitinating enzyme USP14 induces tau elimination in cultured neurons. J. Biol. Chem..

[36] Kiprowska M.J., Stepanova A., Todaro D.R., Galkin A., Haas A., Wilson S.M., Figueiredo-Pereira M.E. (2017). Neurotoxic mechanisms by which the USP14 inhibitor IU1 depletes ubiquitinated proteins and Tau in rat cerebral cortical neurons: Relevance to Alzheimer’s disease. Biochim. Biophys. Acta Mol. Basis Dis..

[37] Xu H., Wang G., Jiang Z., Han Y., Zhao W., Zhang H., Liu H., Liu H., Li Z., Ji F. (2025). Ultrasmall Nanoparticles Mitigate Tau Hyperphosphorylation to Restore Synaptic Integrity and Boost Cognitive Function in Alzheimer’s Disease. Adv. Healthc. Mater..

[38] Minjarez B., Calderón-González K.G., Rustarazo M.L.V., Herrera-Aguirre M.E., Labra-Barrios M.L., Rincon-Limas D.E., Del Pino M.M., Mena R., Luna-Arias J.P. (2016). Identification of proteins that are differentially expressed in brains with Alzheimer’s disease using iTRAQ labeling and tandem mass spectrometry. J. Proteom..

[39] Choi J., Levey A.I., Weintraub S.T., Rees H.D., Gearing M., Chin L.-S., Li L. (2004). Oxidative modifications and down-regulation of ubiquitin carboxyl-terminal hydrolase L1 associated with idiopathic Parkinson’s and Alzheimer’s diseases. J. Biol. Chem..

[40] Xie M., Han Y., Yu Q., Wang X., Wang S., Liao X. (2016). UCH-L1 Inhibition Decreases the Microtubule-Binding Function of Tau Protein. J. Alzheimers Dis..

[41] Zhao Z.B., Wu L., Xiong R., Wang L.L., Zhang B., Wang C., Li H., Liang L., Chen S.D. (2014). MicroRNA-922 promotes tau phosphorylation by downregulating ubiquitin carboxy-terminal hydrolase L1 (UCHL1) expression in the pathogenesis of Alzheimer’s disease. Neuroscience.

[42] Yu Q., Zhang H., Li Y., Liu C., Wang S., Liao X. (2018). UCH-L1 Inhibition Suppresses tau Aggresome Formation during Proteasomal Impairment. Mol. Neurobiol..

[43] Müller U.C., Deller T., Korte M. (2017). Not just amyloid: Physiological functions of the amyloid precursor protein family. Nat. Rev. Neurosci..

[44] Hardy J., Selkoe D.J. (2002). The amyloid hypothesis of Alzheimer’s disease: Progress and problems on the road to therapeutics. Science.

[45] Dufner A., Knobeloch K.-P. (2019). Ubiquitin-specific protease 8 (USP8/UBPy): A prototypic multidomain deubiquitinating enzyme with pleiotropic functions. Biochem. Soc. Trans..

[46] Yeates E.F.A., Tesco G. (2016). The Endosome-associated Deubiquitinating Enzyme USP8 Regulates BACE1 Enzyme Ubiquitination and Degradation. J. Biol. Chem..

[47] Ariyath A., Arigo F.D., Wallach I., Fernando W., Martins R.N., Bharadwaj P. (2026). Novel Small-Molecule Analogues of IU1 Ameliorate Amyloid-β Mediated Toxicity in Alzheimer’s Disease Cell and Worm Models. Int. J. Mol. Sci..

[48] Ariyath A., Hone E., Fernando W.M.A.D.B., Pedrini S., Martins R., Bharadwaj P. (2026). USP14 inhibitor IU1 alleviates amyloid-β mediated toxicity in Alzheimer’s disease cell and worm models. J. Alzheimers Dis..

[49] Zhong B., Liu X., Wang X., Chang S.H., Liu X., Wang A., Reynolds J.M., Dong C. (2012). Negative regulation of IL-17-mediated signaling and inflammation by the ubiquitin-specific protease USP25. Nat. Immunol..

[50] Zheng Q., Song B., Li G., Cai F., Wu M., Zhao Y., Jiang L., Guo T., Shen M., Hou H. (2022). USP25 inhibition ameliorates Alzheimer’s pathology through the regulation of APP processing and Aβ generation. J. Clin. Investig..

[51] Jung E.S., Hong H., Kim C., Mook-Jung I. (2015). Acute ER stress regulates amyloid precursor protein processing through ubiquitin-dependent degradation. Sci. Rep..

[52] Porchietto E., Morello G., Cicilese G., Rainero I., Rubino E., Tamagno E., Boschi S., Guglielmotto M. (2025). UCH-L1 in Alzheimer’s Disease: A Crucial Player in Dementia-Associated Mechanisms. Int. J. Mol. Sci..

[53] Zhang M., Deng Y., Luo Y., Zhang S., Zou H., Cai F., Wada K., Song W. (2012). Control of BACE1 degradation and APP processing by ubiquitin carboxyl-terminal hydrolase L1. J. Neurochem..

[54] Guglielmotto M., Monteleone D., Boido M., Piras A., Giliberto L., Borghi R., Vercelli A., Fornaro M., Tabaton M., Tamagno E. (2012). Aβ1-42-mediated down-regulation of Uch-L1 is dependent on NF-κB activation and impaired BACE1 lysosomal degradation. Aging Cell.

[55] Zhang M., Cai F., Zhang S., Zhang S., Song W. (2014). Overexpression of ubiquitin carboxyl-terminal hydrolase L1 (UCHL1) delays Alzheimer’s progression in vivo. Sci. Rep..

[56] Zhang Z., Yang X., Song Y.-Q., Tu J. (2021). Autophagy in Alzheimer’s disease pathogenesis: Therapeutic potential and future perspectives. Ageing Res. Rev..

[57] Raimondi M., Cesselli D., Di Loreto C., La Marra F., Schneider C., Demarchi F. (2019). USP1 (ubiquitin specific peptidase 1) targets ULK1 and regulates its cellular compartmentalization and autophagy. Autophagy.

[58] Zhao L., Wang X., Yu Y., Deng L., Chen L., Peng X., Jiao C., Gao G., Tan X., Pan W. (2018). OTUB1 protein suppresses mTOR complex 1 (mTORC1) activity by deubiquitinating the mTORC1 inhibitor DEPTOR. J. Biol. Chem..

[59] Lee B., Kim Y.H., Lee W., Choi H.Y., Lee J., Kim J., Mai D.N., Jung S.F., Kwak M.S., Shin J.S. (2023). USP13 deubiquitinates p62/SQSTM1 to induce autophagy and Nrf2 release for activating antioxidant response genes. Free Radic. Biol. Med..

[60] Peng H., Yang F., Hu Q., Sun J., Peng C., Zhao Y., Huang C. (2020). The ubiquitin-specific protease USP8 directly deubiquitinates SQSTM1/p62 to suppress its autophagic activity. Autophagy.

[61] Zhang Z., Chen S., Jun S., Xu X., Hong Y., Yang X., Zou L., Song Y.Q., Chen Y., Tu J. (2025). MLKL-USP7-UBA52 signaling is indispensable for autophagy in brain through maintaining ubiquitin homeostasis. Autophagy.

[62] Basic M., Hertel A., Bajdzienko J., Bonn F., Tellechea M., Stolz A., Kern A., Behl C., Bremm A. (2021). The deubiquitinase USP11 is a versatile and conserved regulator of autophagy. J. Biol. Chem..

[63] Lee J.H., Park S., Kim E., Lee M.J. (2019). Negative-feedback coordination between proteasomal activity and autophagic flux. Autophagy.

[64] Heneka M.T., Carson M.J., El Khoury J., Landreth G.E., Brosseron F., Feinstein D.L., Jacobs A.H., Wyss-Coray T., Vitorica J., Ransohoff R.M. (2015). Neuroinflammation in Alzheimer’s disease. Lancet Neurol..

[65] Wang C., Zong S., Cui X., Wang X., Wu S., Wang L., Liu Y., Lu Z. (2023). The effects of microglia-associated neuroinflammation on Alzheimer’s disease. Front. Immunol..

[66] She L.Y., Li L.Y., Tang H., Yu Q., Gao F.Y., Zeng Y.Q., Chen L.J., Xiong L., Li L.W., Chen F. (2025). OTUD1 positively regulates microglia neuroinflammation and promotes the pathogenesis of Alzheimer’s disease by deubiquitinating C/EBPβ. Acta Pharmacol. Sin..

[67] She L., Li M., Chen F., Wu X., Zhang J., Jiang W., Sun Z., Li L., Ren J., Chen D. (2026). Microglial OTUD6A promotes neuroinflammation and Alzheimer’s disease pathogenesis by deubiquitinating C/EBPβ. Pharmacol. Res..

[68] Sun J., Chen F., She L., Zeng Y., Tang H., Ye B., Zheng W., Xiong L., Li L., Li L. (2025). YOD1 regulates microglial homeostasis by deubiquitinating MYH9 to promote the pathogenesis of Alzheimer’s disease. Acta Pharm. Sin. B.

[69] He D., Zhao H., Zhu Y., Kou Y., Liu T., Huang H., Yang H., Zhang L., Deng J., Xu F. (2026). USP53 promotes NOTCH2-induced neuroinflammation in Alzheimer’s disease. Neurochem. Int..

[70] Ren J., Xiang B., Xueling L., Han X., Yang Z., Zhang M., Zhang Y. (2024). Molecular mechanisms of mitochondrial homeostasis regulation in neurons and possible therapeutic approaches for Alzheimer’s disease. Heliyon.

[71] Sridhar S.K., Goudanavar P., Naveen N.R. (2026). Mitochondrial Dysfunction in Alzheimer’s Disease: Beyond Energy Failure Toward a Pathogenic Nexus. Curr. Alzheimer Res..

[72] Durcan T.M., Tang M.Y., Pérusse J.R., Dashti E.A., Aguileta M.A., McLelland G.L., Gros P., Shaler T.A., Faubert D., Coulombe B. (2014). USP8 regulates mitophagy by removing K6-linked ubiquitin conjugates from parkin. Embo. J..

[73] Mauri S., Bernardo G., Martinez A., Favaro M., Trevisan M., Cobraiville G., Fillet M., Caicci F., Whitworth A.J., Ziviani E. (2023). USP8 Down-Regulation Promotes Parkin-Independent Mitophagy in the Drosophila Brain and in Human Neurons. Cells.

[74] Bernardo G., Prado M.A., Dashtmian A.R., Favaro M., Mauri S., Borsetto A., Marchesan E., Paulo J.A., Gygi S.P., Finley D.J. (2024). USP14 inhibition enhances Parkin-independent mitophagy in iNeurons. Pharmacol. Res..

[75] Chakraborty J., von Stockum S., Marchesan E., Caicci F., Ferrari V., Rakovic A., Klein C., Antonini A., Bubacco L., Ziviani E. (2018). USP14 inhibition corrects an in vivo model of impaired mitophagy. EMBO Mol. Med..

[76] Cornelissen T., Haddad D., Wauters F., Van Humbeeck C., Mandemakers W., Koentjoro B., Sue C., Gevaert K., De Strooper B., Verstreken P. (2014). The deubiquitinase USP15 antagonizes Parkin-mediated mitochondrial ubiquitination and mitophagy. Hum. Mol. Genet..

[77] Wang Y., Serricchio M., Jauregui M., Shanbhag R., Stoltz T., Di Paolo C.T., Kim P.K., McQuibban G.A. (2015). Deubiquitinating enzymes regulate PARK2-mediated mitophagy. Autophagy.

[78] Niu K., Fang H., Chen Z., Zhu Y., Tan Q., Wei D., Li Y., Balajee A.S., Zhao Y. (2020). USP33 deubiquitinates PRKN/parkin and antagonizes its role in mitophagy. Autophagy.

[79] Yu W., Zhuang S., Zhan M., Chen Y., Zhang J., Chen L., Tu C., Zheng L., Chen S. (2024). Deubiquitinating Enzyme USP19 Regulates Ferroptosis and Mitochondrial Damage in SH-SY5Y Cells by Targeting the NOX4 Protein. J. Alzheimers Dis..

[80] Bingol B., Tea J.S., Phu L., Reichelt M., Bakalarski C.E., Song Q., Foreman O., Kirkpatrick D.S., Sheng M. (2014). The mitochondrial deubiquitinase USP30 opposes parkin-mediated mitophagy. Nature.

[81] Kluge A.F., Lagu B.R., Maiti P., Jaleel M., Webb M., Malhotra J., Mallat A., Srinivas P.A., Thompson J.E. (2018). Novel highly selective inhibitors of ubiquitin specific protease 30 (USP30) accelerate mitophagy. Bioorg. Med. Chem. Lett..

[82] Fang T.Z., Sun Y., Pearce A.C., Eleuteri S., Kemp M., Luckhurst C.A., Williams R., Mills R., Almond S., Burzynski L. (2023). Knockout or inhibition of USP30 protects dopaminergic neurons in a Parkinson’s disease mouse model. Nat. Commun..

[83] Jiang Y., Bian W., Chen J., Cao X., Dong C., Xiao Y., Xu B., Sun X. (2023). miRNA-137-5p improves spatial memory and cognition in Alzheimer’s mice by targeting ubiquitin-specific peptidase 30. Anim. Model. Exp. Med..

[84] Corsetti V., Florenzano F., Atlante A., Bobba A., Ciotti M.T., Natale F., Della Valle F., Borreca A., Manca A., Meli G. (2015). NH2-truncated human tau induces deregulated mitophagy in neurons by aberrant recruitment of Parkin and UCHL-1: Implications in Alzheimer’s disease. Hum. Mol. Genet..

[85] Zhang Y., Guo O., Huo Y., Wang G., Man H.Y. (2018). Amyloid-β Induces AMPA Receptor Ubiquitination and Degradation in Primary Neurons and Human Brains of Alzheimer’s Disease. J. Alzheimers Dis..

[86] Gong B., Cao Z., Zheng P., Vitolo O.V., Liu S., Staniszewski A., Moolman D., Zhang H., Shelanski M., Arancio O. (2006). Ubiquitin hydrolase Uch-L1 rescues beta-amyloid-induced decreases in synaptic function and contextual memory. Cell.

[87] Reinitz F., Chen E.Y., di Robilant B.N., Chuluun B., Antony J., Jones R.C., Gubbi N., Lee K., Ho W.H.D., Kolluru S.S. (2022). Inhibiting USP16 rescues stem cell aging and memory in an Alzheimer’s model. eLife.

[88] Guo Y., Cai C., Zhang B., Tan B., Tang Q., Lei Z., Qi X., Chen J., Zheng X., Zi D. (2024). Targeting USP11 regulation by a novel lithium-organic coordination compound improves neuropathologies and cognitive functions in Alzheimer transgenic mice. EMBO Mol. Med..

[89] Harrigan J.A., Jacq X., Martin N.M., Jackson S.P. (2018). Deubiquitylating enzymes and drug discovery: Emerging opportunities. Nat. Rev. Drug Discov..

[90] Wang Y., Jiang Y., Ding S., Li J., Song N., Ren Y., Hong D., Wu C., Li B., Wang F. (2018). Small molecule inhibitors reveal allosteric regulation of USP14 via steric blockade. Cell Res..

[91] Terstappen G.C., Meyer A.H., Bell R.D., Zhang W. (2021). Strategies for delivering therapeutics across the blood-brain barrier. Nat. Rev. Drug Discov..

[92] Lange S.M., Armstrong L.A., Kulathu Y. (2022). Deubiquitinases: From mechanisms to their inhibition by small molecules. Mol. Cell.

